# Serum and tissue miRNAs: potential biomarkers for the diagnosis of cervical cancer

**DOI:** 10.1186/s12985-019-1220-y

**Published:** 2019-10-07

**Authors:** Mahdieh Farzanehpour, Sayed-Hamidreza Mozhgani, Somayeh Jalilvand, Ebrahim Faghihloo, Setareh Akhavan, Vahid Salimi, Talat Mokhtari Azad

**Affiliations:** 1 Department of Virology, School of Public Health, Tehran University of Medical Sciences, Tehran, 1471613151 Iran; 2 Department of Microbiology, School of Medicine, Alborz University of Medical Sciences, Karaj, Iran; 3 Non-communicable Diseases Research Center, Alborz University of Medical Sciences, Karaj, Iran; 4 Department of Microbiology, School of Medicine, Shahid Beheshti University of Medical Sciences, Tehran, Iran; 5 Department of Gynecology Oncology, Imam Khomeini Hospital Complex, Valiasr Hospital, Tehran University of Medical Sciences, Tehran, Iran

**Keywords:** Cervical cancer, Human papillomavirus, miRNA

## Abstract

**Background:**

Finding new biomarkers for the early detection of cervical cancer is an essential requirement in this field. In this study, we aimed to evaluate the expression level of potential biomarkers in progression of cervical cancer in patients with cervical cancer compared to normal subjects.

**Methods:**

The expression levels of tissue and serum miRNAs, including miR-9, miR-192 and miR-205, were investigated in 36 normal, 18 precancer, and 18 cervical cancer samples using real-time polymerase chain reaction.

**Results:**

The results showed the higher significant expressions of miR-9, miR-192 and miR-205 in the tissue of cancer samples than those in the normal samples. Moreover, the miR-192 and miR-205 expression were significantly increased in the cancer group in comparison with the precancer group. Examination of serum samples revealed the increase in the expression level in the cancer groups than in the normal samples, for miR-9, miR-192 and miR-205 and the expressions of miR-9, miR-192 and miR-205 were significantly up-regulated in the precancer group in comparison with the normal group. Also the expression of miR-205 was remarkably increased in the cancer group in comparison with the precancer group. The receiver operating characteristic (ROC) analyses showed the highest area under the curve value for miR-192.

**Conclusions:**

Given the increased expression level of miR-192 in cancer and in precancerous tissue and serum compared with the normal tissue and serum validated by analysing the ROC curve, miR-192 can be used as potential biomarker for the early detection of cervical cancer.

## Background

Cervical cancer is a one of the known fatal cancers among women worldwide, with an estimated death rate of about 60% in the subsequent decade [1, 2]. Human papillomavirus (HPV) is a non-enveloped double-stranded DNA virus that undergoes its life cycle in either the mucosal or the cutaneous stratified squamous epithelia [3]. Twelve HPVs (16, 18, 31, 33, 35, 39, 45, 51, 52, 56, 58 and 59) are defined by the International Agency for Research on Cancer as high-risk (HR) cancer-causing types, with additional types being recognised as ‘possibly’ cancer-causing (68 and 73) [4]. Persistent HR-HPVs are the major aetiological agent in the cervical cancer pathogenesis [5]**.** After HR-HPV infection, diverse cellular changes associated with the epithelial–mesenchymal transition (EMT) are observed [6]. The EMT turns epithelial cells into mesenchymal cells, which can invade and migrate. Moreover, it contributes to the metastatic progression of human cancer cells [7].

About 90% of human genes are predicted to be regulated by miRNAs. MiRNAs may have significant effects on the gene expression in approximately every biological process [8, 9]. Evidence shows that miRNAs are directly involved in cancer pathogenesis; therefore, discovering their expression levels is beneficial to determine the disparate stages of cancer and treatment [10–13].

The expression of multiple miRNAs is dysregulated in various cancers. Moreover, some viruses, particularly oncogenic viruses, can modulate miRNA expressions [14, 15]. The overexpression of miR-9 can lead to EMT, as regulated by c-Myc and Prospero homeobox 1 (PROX1) [16, 17]. It also significantly decreases E-cadherin and increases vimentin through increased cell motility and invasiveness [18]. Moreover, miR-9 promotes EMT and metastasis by directly regulating the KLF17 expression [19].

The miR-192 biological effects are known to some extent. MiR-192 can increase cell proliferation and migration and decrease cell apoptosis and the progression of cellular cells from G0/G1 to stage S by setting the main factors in this progression [20, 21]. Accordingly, miR-192 has been found in a number of cancers, such as colon cancer, colorectal cancer and lung cancer [22, 23]. MiR-192 has been reported to be upregulated in a number of cancers, including gastric cancer, hepatocellular carcinoma, neuroblastoma and adenocarcinoma of the pancreatic modulus [20, 24–26].

MiR-205 is often dysregulated in many cancers, and it serves as a tumour suppressor or an oncogene depending on the cellular base [27]. As an oncogene, miR-205 is significantly overexpressed in the human cervical cancer tissue, and it increases the proliferation and migration of cervical cancer cells by targeting CYR61 and CTGF [28]. To diagnose lung cancer, especially in its early stages, circulating miR-205 was reported as a biological marker [29].

In this study, we investigated for the first time the biomarker potential of a collection of miRNAs among cervical cancer patients. The receiver operating characteristic (ROC) curve analysis was employed to compare the selected groups for the diagnosis of cervical cancer.

## Materials and methods

### Tissue and serum sample collection

Seventy-two fresh uterine cervix biopsies were obtained after hysterectomy and kept in RNAlater (Qiagen) at − 80 °C to stabilise the RNA. The cervical cancer and precancer samples were prepared from the cervical tissues of patients who were from different regions of Iran with informed consent before operation at Imam Khomeini Complex Hospital (Tehran, Iran). The normal samples were obtained from non-cancerous patients. Patients who received any neoadjuvant chemotherapy or intraoperative radiation therapy were excluded. Moreover, subjects had no genetically and sexually transmitted diseases. Women who had maximum three deliveries were included in this study.

The routine haematoxylin–eosin stains on 5 μm paraffin sections were used to make the biopsies at colposcopy and surgery, assessed in the participating hospitals, and then classified as normal, precancer, or invasive cancer according to international criteria [30]. The serum samples were acquired from 5 ml of whole blood specimens of the patients and were immediately frozen at − 80 °C until RNA extraction. This study was approved by the Ethics Committee of Tehran University of Medical Sciences (IR.TUMS.SPH.REC.1395.838).

### miRNA extraction from samples

MicroRNAs were isolated from the serum and tissue using the mirVana™ miRNA Isolation Kit Ambion-1556 (AM1556; Austin, TX, USA) according to the manufacturer’s instructions.

### Stem–loop real-time polymerase chain reaction (RT-PCR) for miRNAs of the tissue and serum

A panel of three miRNAs (miR-9, miR-192 and miR-205) was empirically chosen for RT-PCR analysis in the tissue and serum samples along with their relevant normal controls. The specific stem–loop primers of the miRNAs were designed (Table 1).

**Table 1:** The specific stem-loop primers of miRNAs

Name	Sequence (5′ → 3′)
U6 (forward)	5′-GAGAAGATTAGCATGGCCCCT- 3’
U6 (forward)	5′-ATATGGAACGCTTCACGAATTTGC- 3’
Stem-loop forward RT-miR-9-5P	5′-GTCGTATCCAGTGCAGGGTCCGAGGTATTC GCACTGGATACGACTCATAC-3’
Stem-loop forward RT- miR − 192-5P	5′-GTCGTATCCAGTGCAGGGTCCGAGGTATTC GCACTGGATACGACGGCTGT-3’
Stem-loop forward RT- miR − 205-5P	5′-GTCGTATCCAGTGCAGGGTCCGAGGTATTC GCACTGGATACGACCAGACT-3’
Forward miR − 9-5P	5′-CTTTGGTTATCTAGCTGTATGAGTCGT-3’
Forward miR − 192-5P	5′-CTGACCTATGAATTGACAGCCGT-3’
Forward miR − 205-5P	5′-TCCTTCATTCCACCGGAGTC-3’
Universal reverse	5′-ATCCAGTGCAGGGTCCGA-3’

Briefly, 100 ng of the template RNA (extracted from the tissue and serum) was reverse-transcribed (in 10 μl) using a stem–loop primer. For the PCR reaction, 2 μl of the RT product was used (Applied Biosystems, Foster City, CA, USA). The threshold for the presence of miRNAs was considered as the C_t_-value (threshold cycle) lower than 30. The U6 snRNA was used as the housekeeping gene.

### Quantitative RT-PCR (qRT-PCR) analysis

The expressions of miR-9, miR-192 and miR-205 were analysed by qRT-PCR using the SYBR Green (Takara Bio Inc., Otsu, Japan) on an Applied Biosystems®StepOnePlus™ RT-PCR system (Applied Biosystems, Foster City, CA, USA). To normalise the relative quantity, U6 snRNA was performed for the miRNAs [31, 32].

### Nested PCR for HPV typing

DNA was extracted with the High Pure DNA extraction kit (Roche, Germany). To check the integrity of the extracted DNA and the absence of PCR inhibitors, a 110 bp segment of the human β-globin gene was amplified by PC03/PC04 primers [33]. The detection of HPV sequences was conducted by two sets of consensus primers, MY09/MY11 and GP5+/GP6+ [33], which amplify a 450 bp and an internal 150 bp region, respectively, in the highly conserved L1 HPV gene. After amplification, the reaction products were electrophoresed on 2% agarose and visualised by SYBR Safe dye.

### DNA sequencing and genotyping

ABI (Applied Biosystems) BigDye 3.1 Chemistry was used for cycle sequencing. Double-stranded sequencing runs were performed on ABI 3730 sequencers with 50 cm capillaries. The most conserved gene of L1 ORF was used to identify the new HPV types. The sequencing outcomes for each patient were collected and aligned with each other using BioEdit software (http://www.mbio.ncsu.edu/bioedit/bioedit.html). The subtyping was conducted by Basic Local Alignment Search Tool (BLAST) analysis with reference sequences, from the GenBank database (NCBI).

### Statistical analysis

The Mann–Whitney non-parametric test and the one-way ANOVA were performed to analyse the statistical discrepancy among the groups using GraphPad Prism (7.0.1) software. A *p*-value less than 0.05 was considered significant. The area under the ROC curves was calculated using R software (version 3.4.4).

## Results

### Patient and control data

Seventy-two fresh samples, including 36 normal, 18 precancer and 18 cervical cancer, were prepared for the miR-9, miR-192 and miR-205 expression analyses. The mean ages of the cervical cancer, precancer and normal groups were 61 (range: 45–81), 47 (range: 27–57) and 36 (range: 23–49), respectively. No significant discrepancy was found among the three groups.

### miRNA expression profiles in the tissue samples

**Fig. 1:**
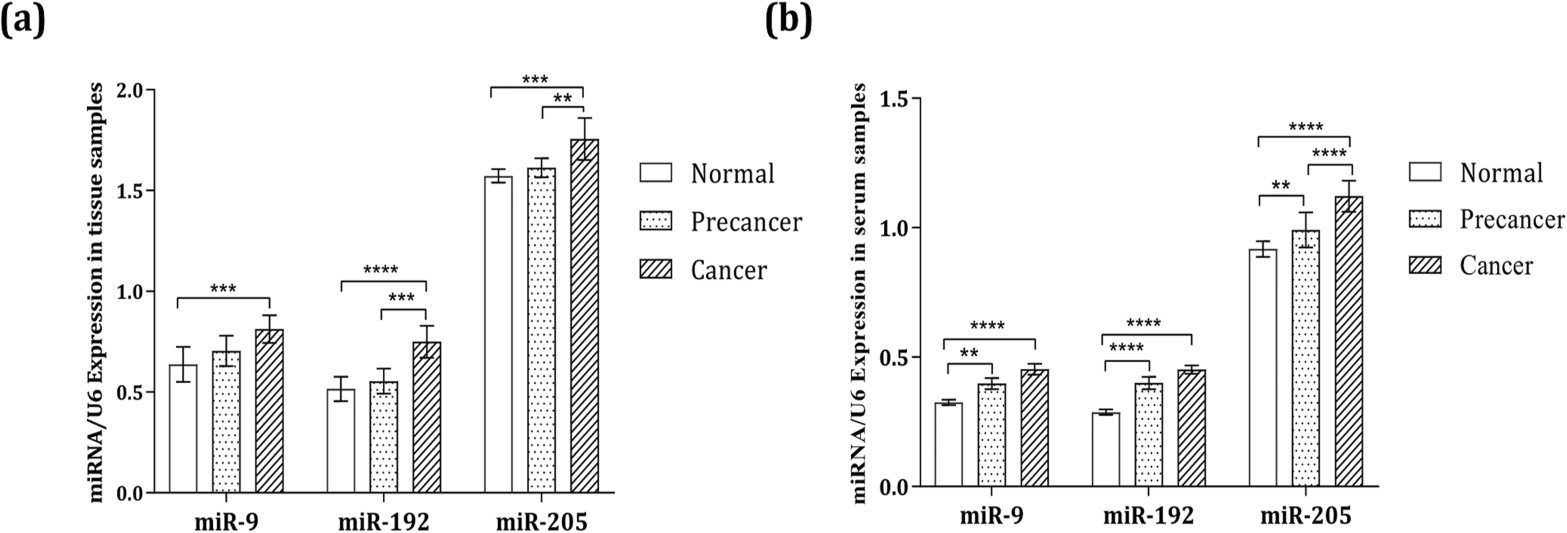
Relative expression levels of miR-9, miR-192 and miR-205 among the cervical cancer, precancer and normal groups in the (**a**) tissue and (**b**) serum samples. Each sample was analysed in triplicate and normalised to U_6_

The results showed the higher significant expressions of miR-9, miR-192 and miR-205 in the tissue of cancer samples than those in the normal samples, with *p*-values of 0.0008, < 0.0001 and 0.0005, respectively. Moreover, the miR-192 and miR-205 expression were significantly increased in the cancer group in comparison with the precancer group, with a *p*-values of 0.0002 and 0.0009 (Fig. 1a).

### miRNA expression profiles in the serum samples

The serum expression levels of miR-9, miR-192 and miR-205 were analysed. The outcomes revealed the increase in the expression level in the cancer groups than in the normal samples, with *p*-values of < 0.0001 for miR-9, < 0.0001 for miR-192 and < 0.0001 for miR-205. The expressions of miR-9, miR-192 and miR-205 were significantly up-regulated in the precancer group in comparison with the normal group, with p-values of 0.005, < 0.0001 and 0.005, respectively. The expression of miR-205 was markedly increased in the cancer group in comparison with the precancer group, with a *p*-value of < 0.0001 (Fig. 1b).

### Correlation analysis between the potential biomarkers

To comprehend the associations among the miRNA expressions, the correlation values were analysed. The meaningful associations were observed between miR-9 and miR-192 (r = 0.66, *p*-value = 0.004) and between miR-192 and miR-205 (r = 0.68, *p*-value = 0.002) in the serum specimens of the cancer group. The nearly significant correlations were obtained between miR-9 and miR-205 (r = 0.13, p-value < 0.0001), between miR-9 and miR-192 (r = 0.08, p-value < 0.0001) and between miR-192 and miR-205 (r = 0.71, p-value = 0.001) in the serum samples of the precancer groups. A significant correlation was found between miR-9 samples and miR-192 in the tissue samples (r = 0.66, *p*-value = 0.004).

### ROC curve analysis

**Fig. 2:**
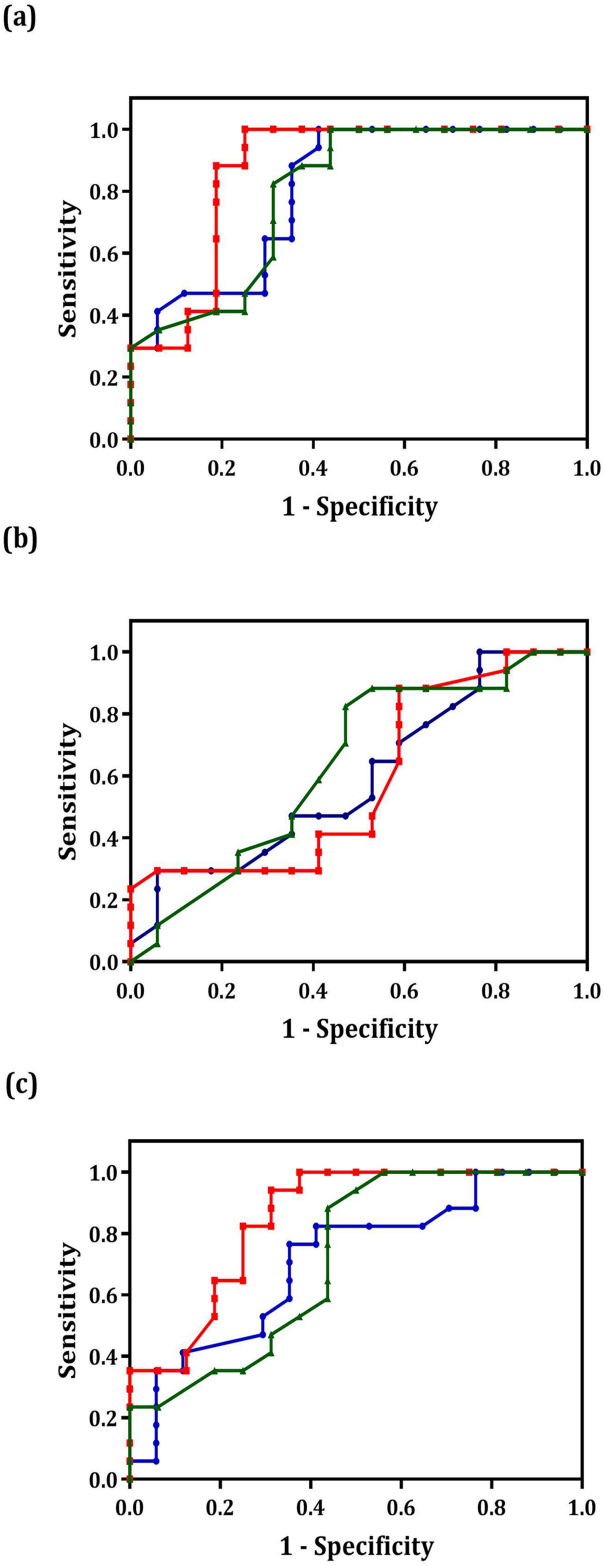
ROC curve analysis using miR-9, miR-192 and miR-205 to distinguish the different groups in the tissue samples. The area under the curve (AUC) values in the cervical cancer and normal groups (**a**), the AUC values in the precancer and normal groups (**b**) and The AUC values in the cervical cancer and precancer groups (**c**). The red, blue and green lines represent miR-192, miR-9 and miR-205, respectively

**Fig. 3:**
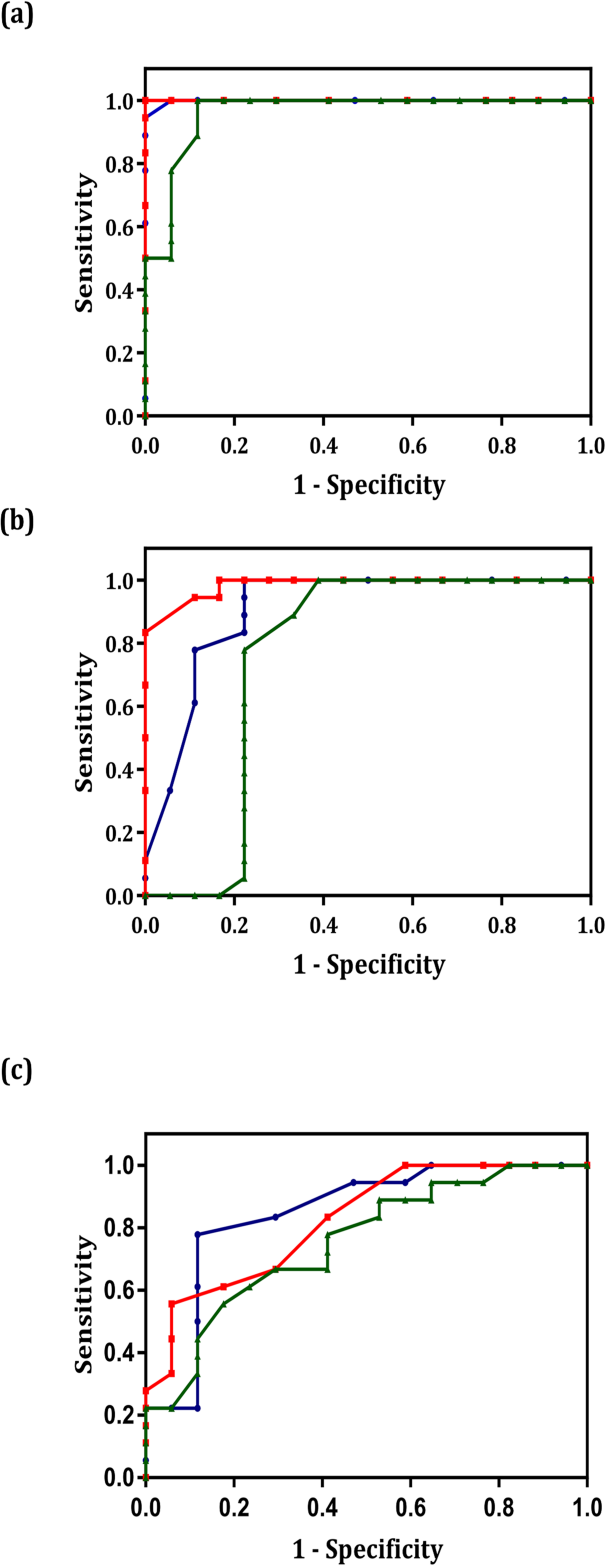
ROC curve analysis using miR-9, miR-192 and miR-205 to distinguish the different groups in the serum samples. The area under the curve (AUC) values in the cervical cancer and normal groups (**a**), the AUC values in the precancer and normal groups (**b**) and The AUC values in the cervical cancer and precancer groups (**c**). The red, blue and green lines represent miR-192, miR-9 and miR-205, respectively

The ROC curves were generated, and their under area analyses were conducted to evaluate the diagnostic value of the miR-9, miR-192 and miR-205 expression levels in the tissue samples of the cervical cancer, precancer and normal groups (Table 2). The ROC curves revealed that the area under the curve (AUC) values in the cervical cancer and normal groups were miR-9: 0.80 (95% CI: 0.66–0.95), miR-192: 0.87 (95% CI: 0.73–1) and miR-205: 0.79 (95% CI: 0.64–0.95) (Fig. 2a). The AUC values in the precancer and normal groups were miR-9: 0.59 (95% CI: 0.40–0.79), miR-192: 0.59 (95% CI: 0.39–0.78) and miR-205: 0.64 (95% CI: 0.44–0.82) (Fig. 2b). The AUC values in the cervical cancer and precancer groups were miR-9: 0.71 (95% CI: 0.53–0.89), miR-192: 0.85 (95% CI: 0.71–0.98) and miR-205: 0.71 (95% CI: 0.54–0.90) (Fig. 2c, Table 3). The same ROC analyses were performed for the serum samples (Table 4). The ROC curves showed that the AUC values in the cervical cancer and normal groups were miR-9: 0.99 (95% CI: 0.99–1), miR-192: 1 (95% CI: 1–1) and miR-205: 0.96 (95% CI: 0.89–1) (Fig. 3a). The AUC values in the precancer and normal groups were miR-9: 0.90 (95% CI: 0.80–1), miR-192: 0.98 (95% CI: 0.95–1) and miR-205: 0.75 (95% CI: 0.56–0.95) (Fig. 3b). The AUC values in the cervical cancer and precancer groups were miR-9: 0.85 (95% CI: 0.71–0.98), miR-192: 0.82 (95% CI: 0.69–0.96) and miR-205: 0.75 (95% CI: 0.59–0.91) (Fig. 3c). The highest AUC value was obtained for miR-192 in both the serum and tissue samples from comparing the cervical cancer and the normal groups. MiR-192 had a strong potential diagnosis value for differentiating the cervical cancer groups from the normal groups. However, other results also confirmed that miR-9 and miR-205 could have such potential. Moreover, the sensitivity and the specificity of miR-192 were higher than those of the two other microRNAs in the comparison between the cervical cancer and normal groups at 75 and 94.1% in the tissue samples and 100.0 and 94.4% in the serum samples, respectively. The same results in specificity were obtained for miR-9. Other sensitivity and specificity values are presented in Tables 4 and 5.

**Table 2:** ROC curve analysis. Area under the curve (AUC) value of miR-9, miR-192, and miR-205 in tissue samples

miRNA	Cervical cancer and Normal groups	Cervical cancer and Precancer cervical groups	Precancer cervical and Normal groups
miR-9	AUC 95% CI: 0.80 (0.66–0.95)	AUC 95% CI: 0.71 (0.53–0.89)	AUC 95% CI: 0.59 (0.40–0.79)
miR-192	AUC 95% CI: 0.87 (0.73–1)	AUC 95% CI: 0.85 (0.71–0.98)	AUC 95% CI: 0.59 (0.39–0.78)
miR-205	AUC 95% CI: 0.79 (0.64–0.95)	AUC 95% CI: 0.71 (0.54–0.90)	AUC 95% CI: 0.64 (0.44–0.82)

**Table 3:** The sensitivity and specificity estimation of miR-9, miR-192, and miR-205 according to the ROC curves results in tissue samples

miRNA	Sensitivity and Specificity	Cervical cancer and Normal groups	Cervical cancer and Precancer cervical groups	Precancer cervical and Normal groups
miR-9	Sensitivity	58.8	64.7	23.5
	Specificity	94.1	76.4	94.1
miR-192	Sensitivity	75	67.7	41.1
	Specificity	94.1	94.1	88.2
miR-205	Sensitivity	56.2	50	47
	Specificity	94.1	94.1	88.2

**Table 4:** ROC curve analysis. Area under the curve (AUC) value of miR-9, miR-192, and miR-205 in the serum samples

miRNA	Cervical cancer and Normal groups	Cervical cancer and Precancer cervical groups	Precancer cervical and Normal groups
miR-9	AUC 95% CI: 0.99 (0.99–1)	AUC 95% CI: 0.85 (0.71–0.98)	AUC 95% CI: 0.90 (0.80–1)
miR-192	AUC 95% CI: 1 (1–1)	AUC 95% CI: 0.82 (0.69–0.96)	AUC 95% CI: 0.98 (0.95–1)
miR-205	AUC 95% CI: 0.96 (0.89–1)	AUC 95% CI: 0.75 (0.59–0.91)	AUC 95% CI: 0.75 (0.56–0.95)

**Table 5:** The sensitivity and specificity estimation of miR-9, miR-192, and miR-205 according to the ROC curves results in serum samples

miRNA	Sensitivity and Specificity	Cervical cancer and Normal groups	Cervical cancer and Precancer cervical groups	Precancer cervical and Normal groups
miR-9	Sensitivity	100	52.9	77.8
	Specificity	94.4	94.4	94.4
miR-192	Sensitivity	100	58.8	83.3
	Specificity	94.4	83.3	94.4
miR-205	Sensitivity	88.2	35.3	66.7
	Specificity	88.9	94.4	88.9

### HPV typing

The molecular typing revealed the presence of HPV DNA in 44.4% of the cases (32/72), with a predominance of HPV type 16 (Table 6). Stratification of the pathological status showed that HPV 16 was found in all samples (100%) in the cancer group; HPV 16 and 53 were detected at 50 and 5.5%, respectively, in the precancer group; and HPV 16, 66 and 68 were identified at 5.5, 2.7 and 2.7%, respectively, in the normal group.

**Table 6:** The distributions of the HPV genotypes

Groups	Virus genotype	Percent and number
Cancer (18)	HPV-16	18 (100%)
Precancer (18)	HPV-16	9 (50%)
	HPV-53	1 (5%)
Normal (36)	HPV-16	2 (5.5%)
	HPV-66	1 (2.7%)
	HPV-68	1 (2.7%)

## Discussion

The molecular identification of new biomarkers for the prognosis of cervical cancer is noteworthy to diagnose the cancers before the malignancy of the tumours [34]. The infection of cervical cancer cells by HPV results in many cellular changes, which can be examined to attain this purpose. In this study, the higher levels of miR-9, miR-192 and miR-205 in the serum and tissue of the cervical cancer and precancer samples infected by HPV were detected in comparison with those in the normal cases. These outcomes suggested that the aforementioned miRNAs could be traced to detect the cervical cancer cells.

MiRNAs belong to a novel category of non-coding RNA molecules that interconnect to target mRNA to degrade or translate repression. Their detection opened a new intuition to the perception of intricate gene regulatory networks. To date, more than 650 miRNAs have been recognised in human cells [35]**.** The mean half-life of the miRNAs in mammalian cells is about five days, which is 10-fold more than the regular mRNAs [36]. miRNAs are highly stable against degradation [37], excessive pH, high temperature and freeze and thaw cycles [38]. These features render miRNAs to be considered as prognostic biomarkers of cancers, which differentiate the normal from tumour tissues [39]. The high sensitivity and specificity and the possibility of the non-invasive collection of miRNAs led to the interest in examining miRNAs as biomarkers [40]. For this purpose, the miR-9, miR-192 and miR-205 levels were investigated in cervical samples.

Various roles were ascribed to miR-9 in various tissues. MiR-9 is involved in the promotion or suppression of metastasis depending on the tissue context. Liu et al. showed the activation of miR-9 by HPV in cervical cancer and suggested that it could be due to the great chromosomal instability caused by the HPV infection [41, 42]. Consistent with other reports [28, 41], our study revealed the up-regulation of miR-9 by HPV in the serum and tissues of the cancer and precancer specimens in comparison with the normal cells.

MiR-205 acts as a proliferation and invasion inhibitor in breast, glioma and oesophageal cancers. Conversely, it simplifies the tumour activation and the proliferation of bladder, lung, head and ovarian cancer cell lines [27, 43]. Lebanony et al. introduced miR-205 as a precise marker to discern non-squamous from squamous non-small-cell lung carcinoma [44]. Moreover, the risk of lymph node metastasis was prognosticated in colorectal cancer tissue by the downregulation of miR-205 expression [45]. Moreover, miR-205 and let-7 were considered diagnostic biomarkers for ovarian cancer [29, 46]. In 2012, the function of miR-205 was introduced as the promotion of the proliferation and migration of cervical cancer cells [28]. Conversely, miR-192 suppresses the ZEB2 and VEGFA expressions in colon cancer cells [47]. Wu et al. showed that miR-192 acts as a regulator of angiogenesis and inhibits the tumour angiogenesis in several renal and ovarian tumour models [48]. They found that ZEB2 and RhoA are the two main targets of miR-192, which serves as the mediator in the process of TGF-β-induced EMT. Wang et al. demonstrated the downregulation of miR-192 in bladder cancer patients [49]. By contrast, Chen et al. reported high levels of miR-192 in the plasma of distant metastasis/gastric cancer samples [50].

Some miRNAs, including miR-9, miR-192 and miR-205, can directly regulate the E-cadherin-encoding mRNA. Moreover, they can have roles in determining final E-cadherin level by binding to its inhibitory factors, such as transcription factors, zinc finger E-box-binding proteins 1 and 2 (ZEB1 and ZEB2) and Snail1 levels [51–53].

In this research, we used the ROC curves to investigate the predictive power of miRNAs as a diagnostic biomarker for cervical cancer. The highest AUC value was found in miR-192: 1 (95% CI: 1–1) in the comparison between the cervical cancer and normal groups, with a sensitivity of 0.75 and 100.0% in the tissue and serum samples, respectively. The nearly highest AUC value and sensitivity were obtained in the comparison between the precancer cancer and normal groups and between the precancer groups and the cancer groups. Therefore, the expression level of miR-192 in the tissue and serum can be tracked for the prognosis and diagnosis of cervical cancer.

The molecular typing revealed the presence of HPV DNA in 44.4% (32/72) of cases with a predominance of HPV 16. It is in agreement with previous reports that showed higher prevalence of HPV 16 [54–58].

Given the importance of microRNAs in preventing or inducing cervical cancer or their potential role in the early detection of disease, many studies have been conducted to identify or measure their expression [59–61]. In a systematic review by Pardini et al., miR-29a and miR-21, cited as the most common down- and up-regulated in invasive cervical cancer (ICC) progression, respectively [62]. Microarray-based studies also showed that miR-10a, miR-20b, miR-9, miR-16 and miR-106 repeatedly dysregulated. In addition, miR-34a, miR-125 and miR-375 were also found dysregulated in cervical exfoliated cells in relation to cancer progression [62].

Limitations of this type of studies include low sample size, possible selection bias and use of convenience material which can affect the final result of miRNAs relevant for cervical cancer. Today, the use of High-throughput Platforms such as microarrays or next-generation sequencing (NGS) can evaluate the MicroRNAs involved in the disease and determined their differences in expression. More accurate results can be obtained using these platforms and formulating studies with appropriate criteria.

## Conclusion

The expression of a significant number of oncogenic and tumour-suppressive miRNAs changes in cervical cancer cells. The cognition of these miRNAs can help to find the key factors and risks for cancer prognosis and treatment. In this study we found that the up-regulation of miR-192 in the serum and tissue is a potential biomarker for the diagnosis of HPV-associated cervical cancer. Therefore, our results can help to identify the possible biomarker for HPV-induced cancers.

## Data Availability

All relevant data are within the paper.

## Abbreviations

AUC: Area under the curve
EMT: Epithelial–mesenchymal transition
HPV: Human papillomavirus
HR: High-risk
PROX1: Prospero homeobox 1
ROC: Receiver operating characteristic
